# Robust Audio–Visual Speaker Localization in Noisy Aircraft Cabins for Inflight Medical Assistance

**DOI:** 10.3390/s25185827

**Published:** 2025-09-18

**Authors:** Qiwu Qin, Yian Zhu

**Affiliations:** School of Computer Science, Northwestern Polytechnical University, Xi’an 710129, China; qqw001@mail.nwpu.edu.cn

**Keywords:** active speaker localization, audio–visual fusion, aircraft cabin environments, in-flight medical scenarios, aviation AI

## Abstract

Active Speaker Localization (ASL) involves identifying both who is speaking and where they are speaking from within audiovisual content. This capability is crucial in constrained and acoustically challenging environments, such as aircraft cabins during in-flight medical emergencies. In this paper, we propose a novel end-to-end Cross-Modal Audio–Visual Fusion Network (CMAVFN) designed specifically for ASL under real-world aviation conditions, which are characterized by engine noise, dynamic lighting, occlusions from seats or oxygen masks, and frequent speaker turnover. Our model directly processes raw video frames and multi-channel ambient audio, eliminating the need for intermediate face detection pipelines. It anchors spatially resolved visual features with directional audio cues using a cross-modal attention mechanism. To enhance spatiotemporal reasoning, we introduce a dual-branch localization decoder and a cross-modal auxiliary supervision loss. Extensive experiments on public datasets (AVA-ActiveSpeaker, EasyCom) and our domain-specific AirCabin-ASL benchmark demonstrate that CMAVFN achieves robust speaker localization in noisy, occluded, and multi-speaker aviation scenarios. This framework offers a practical foundation for speech-driven interaction systems in aircraft cabins, enabling applications such as real-time crew assistance, voice-based medical documentation, and intelligent in-flight health monitoring.

## 1. Introduction

Active Speaker Detection (ASD) is a core task in understanding audio–visual scenes that aims to determine who is speaking in multi-person video scenes. Traditional approaches typically rely either on visual cues—such as lip motion and facial expressions [1]—or on audio-based voice activity detection [2,3,4]. However, visual-only methods may confuse non-verbal mouth movements (e.g., yawning, grimacing) with speech, while audio-only techniques often degrade under noisy or overlapping speech conditions. These limitations underscore the necessity of leveraging both modalities to achieve reliable speaker detection.

Recent advances in multimodal ASD have shown that fusing audio and visual streams markedly improves robustness, especially under real-world degradations. Attention-style fusion [5,6,7] and beamforming-driven spatial reasoning [8] jointly map vocal patterns to dynamic facial cues, while self-supervised pre-training further enhances noise tolerance. Large-scale resources, such as AVA-ActiveSpeaker [9] and the egocentric EasyCom corpus [10], provide the data diversity needed to generalize across domains.

Beyond identifying *who* is speaking, *Active Speaker Localization (ASL)* seeks to pinpoint *where* the speaker is situated in the scene. Audio–visual talker-localization networks [11] and cross-modal DoA tracking frameworks [7,8] demonstrate that tightly coupled spatial cues can yield centimeter-level accuracy under reverberant conditions.

Aviation scenarios—particularly within the confined, acoustically complex environment of an aircraft cabin—raise the stakes for ASL. During in-flight medical emergencies, flight attendants, medical professionals, and passengers must exchange time-critical instructions. Accurate speaker localization can support intelligent crew-assistance systems and post-incident analytics. Yet cabin interiors expose algorithms to challenging conditions: engine noise levels typically range from 74 to 85 dB(A), with signal-to-noise ratios (SNRs) often dropping below 10 dB in wide-body aircraft during cruise [12,13]. Additionally, visual occlusions occur frequently, with studies indicating that up to 30% of video frames in cabin settings may feature partial or complete facial obstructions due to passenger movement or cramped seating arrangements [14]. Fluctuating illumination, including low-light “night mode” conditions, further complicates visual processing [15].

Conventional microphone-array pipelines suffer from spatial aliasing and reverberant reflections, while visual-only cues are hampered by masks, cramped camera angles, and low-light conditions. These constraints motivate **multimodal ASL frameworks** that learn to fuse spatial acoustics with visual dynamics for resilient localization in constrained cabins.

Motivated by these needs, we propose an end-to-end audio--visual ASL framework tailored for in-flight medical communication. The model consumes raw video frames and multi-channel cabin audio, and directly regresses 3D speaker coordinates. Modality-specific encoders feed a cross-attention fusion core, followed by a spatial regression head. Joint objectives encourage lip–speech synchrony, spatial consistency, and noise robustness.

In summary, our main contributions are as follows:

- Re-framing ASL within aviation–medicine workflows, highlighting cabin-specific operational demands;
- Designing a unified, fully end-to-end multimodal localization network that bypasses separate face detection or VAD stages;
- Proposing training objectives and evaluation protocols aligned with high-noise, low-light, occlusion-prone cabin environments;
- Demonstrating state-of-the-art localization accuracy on real and simulated in-flight recordings.

## 2. Related Work

Active Speaker Detection (ASD) has become a foundational task in multimodal perception, supporting applications, such as context-aware transcription, real-time communication monitoring, and intelligent human–machine collaboration [16,17,18,19,20]. Early methods primarily relied on visual features—such as lip motion and facial expressions—extracted via hand-crafted descriptors or convolutional neural networks [21,22]. However, vision-only approaches are often unreliable in constrained environments like aircraft cabins, where lighting conditions fluctuate, faces may be partially occluded by seatbacks or oxygen masks, and available camera views are limited [5].

On the other hand, audio-only localization techniques—such as Time Difference of Arrival (TDoA), beamforming, or subspace methods like MUSIC and MVDR—have shown competitive results in structured indoor settings, such as meeting rooms [23,24]. Yet, these methods degrade significantly under the acoustically reflective and noise-rich conditions present in aircraft interiors. Engine hum, airflow systems, public announcements, and cabin-specific reverberation profiles all introduce complex distortions that hinder reliable direction-of-arrival estimation [25]. Recent studies on adaptive beamforming [26] highlight that dynamically leveraging multi-channel spatial cues can enhance localization robustness, which is highly relevant for the acoustic branch of CMAVFN operating in high-noise aviation environments.

These limitations have prompted increased interest in audio–visual fusion techniques that exploit the complementary strengths of both modalities. Recent works, such as Visually Supervised Speaker Detection and Localization via Microphone Array [24], demonstrate that visual pseudo-labels can effectively supervise spatial audio learning, reducing dependence on clean signals or extensive sensor calibration. Other models adopt attention-based cross-modal encoders or spatio-temporal synchronization to enhance localization accuracy and robustness [5,27]. However, most of these models are trained and evaluated under controlled laboratory conditions or in static meeting scenarios, which lack the dynamic and unpredictable characteristics of airborne medical emergencies.

In aviation-specific settings, research on multimodal communication analysis remains scarce. While some studies have explored cockpit audio processing and cabin surveillance for safety monitoring, few have tackled speaker localization in the context of real-time, multi-party medical interaction. Emergencies during flight present unique challenges: limited spatial freedom for cameras and microphones, moving subjects (e.g., attending crew or assisting passengers), high ambient noise levels, and unpredictable visual occlusion due to cabin geometry and movement. Furthermore, the necessity for hands-free, speech-driven assistance systems—especially when treating unconscious or mobility-impaired passengers—calls for robust ASL mechanisms capable of identifying and spatially grounding active speakers without relying on uninterrupted facial visibility. Recent ideas, such as the Occlusion Sensitivity Parameter (OSS) [28], provide inspiration for improving audio-visual model generalization under severe occlusion conditions, which frequently occur in cabins due to oxygen masks or medical tools.

Previous medical-domain efforts have examined sound-based event recognition in hospital settings [29] and speech detection in ICUs to separate patient utterances from alarm noise [30], validating the broader applicability of acoustic monitoring. However, these approaches often neglect visual signals and do not address the problem of spatial localization of individual speakers, which is essential for real-time interventions and retrospective analysis in high-stakes environments like aircraft cabins.

Moreover, the aviation context introduces several under-addressed constraints: variable camera installation points, changes in cabin lighting (e.g., dimmed mode), multi-language interactions, and rapid changes in the speaker pool. These factors expose the limitations of existing ASD/ASL systems, which are typically developed and evaluated in controlled environments with stable viewpoints, consistent lighting, and predictable speaker dynamics. Such assumptions do not hold in the constrained and rapidly changing conditions of an aircraft cabin, leading to significant degradation in model performance.

Recent research [19,20] has highlighted the potential of combining deep learning with probabilistic modeling for robust multi-speaker tracking. We leverage this approach in the context of aeromedical care by (1) integrating spatially aware audio-processing techniques to accommodate the acoustic characteristics of aircraft cabins; (2) adapting visual encoders to handle partial visibility and suboptimal viewing angles; and (3) implementing a tightly integrated end-to-end fusion architecture capable of stable operation to support reliable speaker localization during in-flight medical emergencies.

## 3. Method

To tackle the challenge of active speaker localization in acoustically and visually constrained environments, such as aircraft cabins during in-flight medical emergencies, we propose a novel cross-modal audio–visual fusion framework tailored to the specific characteristics of airborne scenarios. Unlike static clinical rooms, aircraft cabins exhibit unique constraints: limited installation space for cameras and microphones, frequent occlusion due to passenger movement or medical procedures, and persistent background noise from engines, ventilation systems, and onboard announcements. Our method addresses these challenges by leveraging structured multi-channel cabin audio and spatially localized visual cues, fused via a cross-modal attention mechanism to enable robust, real-time speaker localization.

The overall architecture centers on a Cross-Modal Audio-Visual Fusion Network (CMAVFN), integrating a Cross-Modal Attention Fusion (CM-AF) module and a spatial-temporal prediction network optimized for deployment in airborne medical settings. The following sections describe each component in detail.

### 3.1. Overall Framework

**Figure 1:**
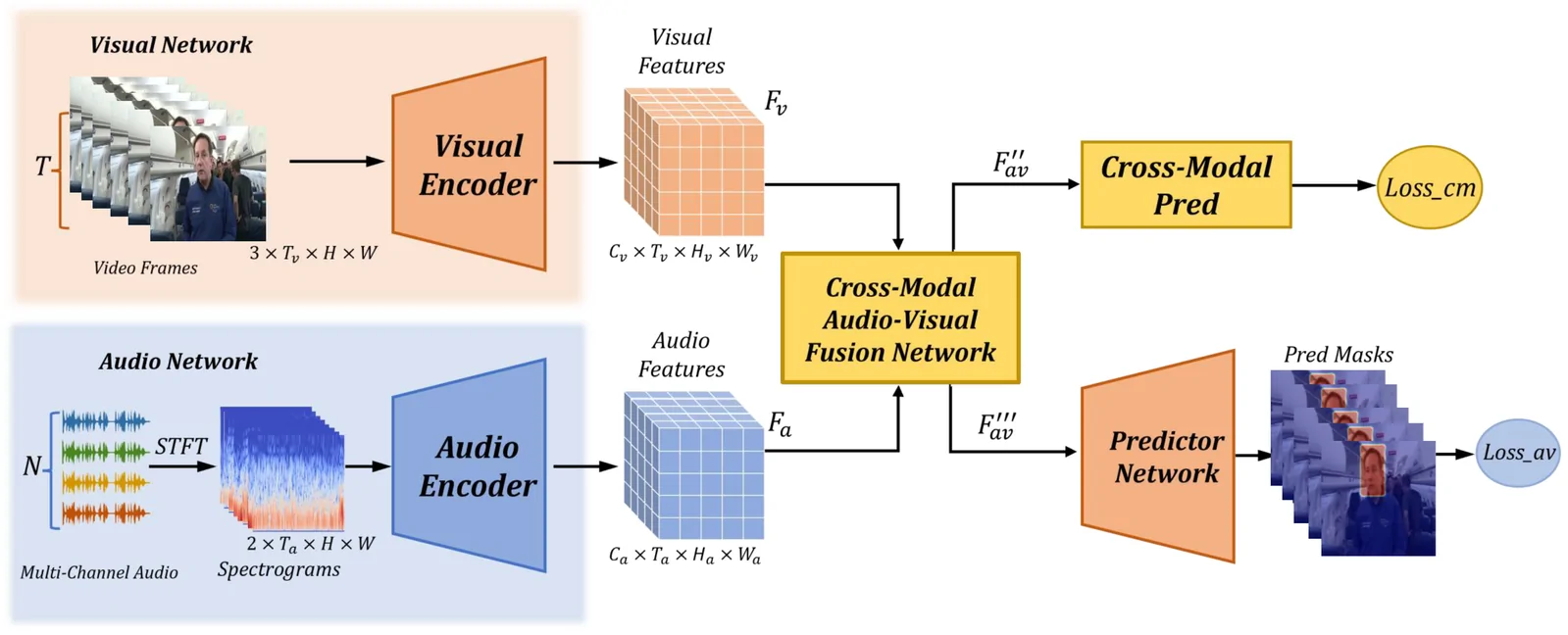
Model architecture of the attention-based cross-modal active speaker localization network.

As illustrated in Figure 1, our proposed pipeline consists of visual and audio encoders, a cross-modal attention fusion module, a dual-branch localization decoder, and a multi-level training objective. Each frame captured by a fixed-angle overhead cabin camera is processed by a visual encoder to extract spatial features $F_{v}$. Simultaneously, time-synchronized six-channel audio collected from compact cabin-mounted microphones undergoes Short-Time Fourier Transform (STFT) and is encoded into $F_{a}$, capturing directionality and speaker-specific acoustic cues.

These unimodal representations are fused via a cross-modal attention mechanism, yielding joint features $F_{av}^{\prime \prime \prime }$ that integrate both modalities over space and time. The fused features are decoded through two branches: a primary prediction head that produces a spatial–temporal speaker localization map, and an auxiliary branch that leverages intermediate features $F_{av}^{\prime \prime }$ for additional supervision. The training objective combines a main loss $Loss_{av}$ and a cross-modal consistency loss $Loss_{cm}$, as defined in Equation (8).

### 3.2. Cross-Modal Audio–Visual Fusion Network

**Figure 2:**
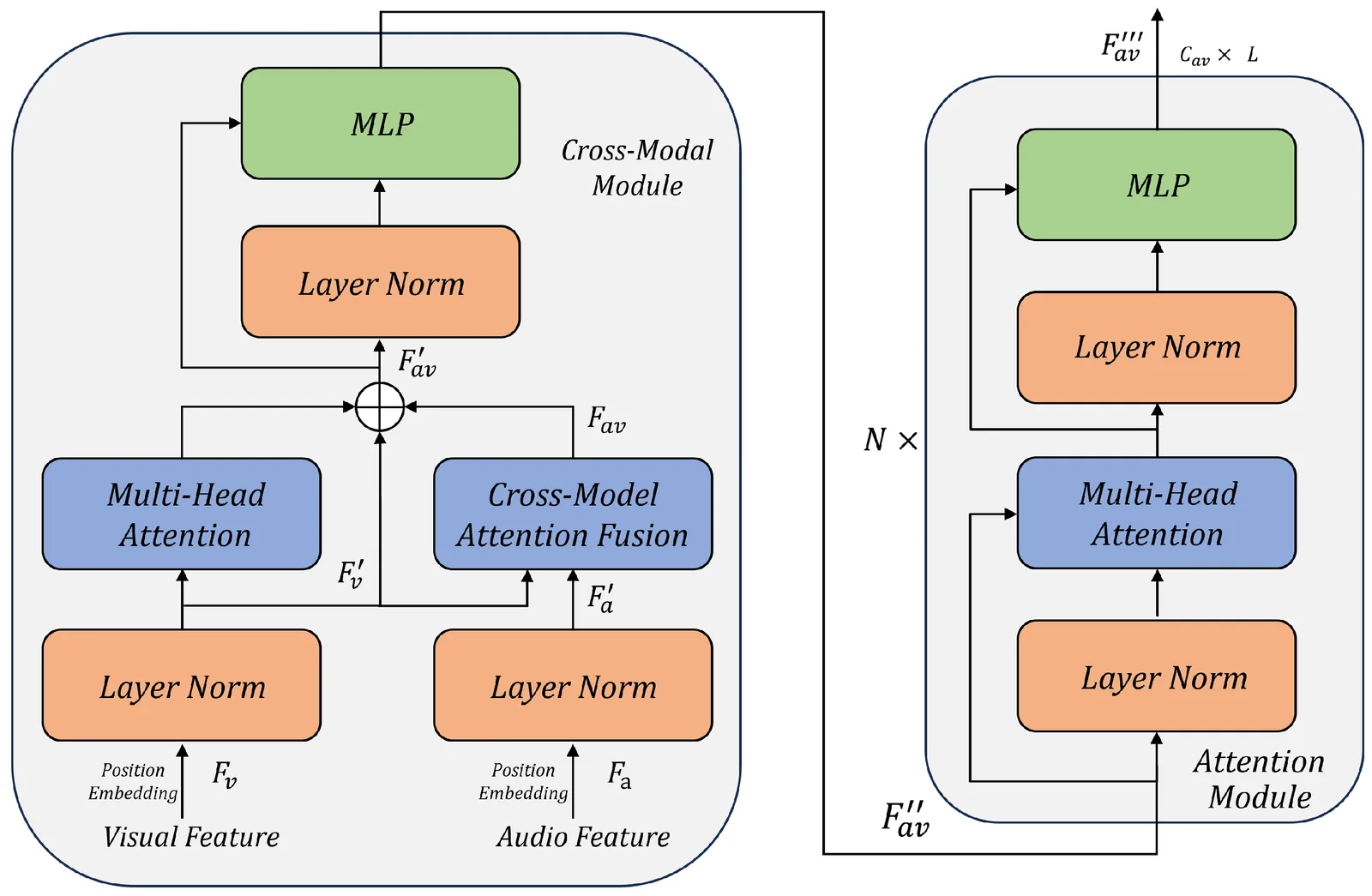
Overall architecture of the Cross-Modal Audio–Visual Fusion Network (CMAVFN).

Aircraft cabins present multimodal perception challenges due to confined geometry, low-frequency engine noise, and variable lighting. In addition, visual occlusions occur frequently due to emergency medical equipment, crew activity, or passenger postures. To address these conditions, our CMAVFN adopts an asymmetric fusion strategy where spatially fine-grained visual features guide the integration of context-rich but noisy audio cues as shown in Figure 2.

We begin by applying positional encoding and normalization:

(1) $$\begin{array}{cc} F_{v}^{\prime } & =\mathrm{Norm}1(F_{v}+PE\left(F_{v}\right))\\ F_{a}^{\prime } & =\mathrm{Norm}1(F_{a}+PE\left(F_{a}\right)) \end{array}$$

We then apply intra- and inter-modal attention:

(2) $$F_{av}^{\prime }=F_{v}^{\prime }+\mathrm{DropPath}\left(\mathrm{Attn}\left(F_{v}^{\prime }\right)+\mathrm{CrossM}\left(F_{v}^{\prime },F_{a}^{\prime }\right)\right)$$

To enhance representation capacity, a residual MLP block further refines the fused features:

(3) $$F_{av}^{\prime \prime }=F_{av}^{\prime }+\mathrm{DropPath}\left(\mathrm{MLP}\left(\mathrm{Norm}2\left(F_{av}^{\prime }\right)\right)\right)$$

This design enables effective modeling of micro facial cues (e.g., speaking under masks or oxygen cannulas) and subtle inter-microphone variations, both critical for operating in noisy, occluded airborne environments.

### 3.3. Cross-Modal Attention Fusion Module

**Figure 3:**
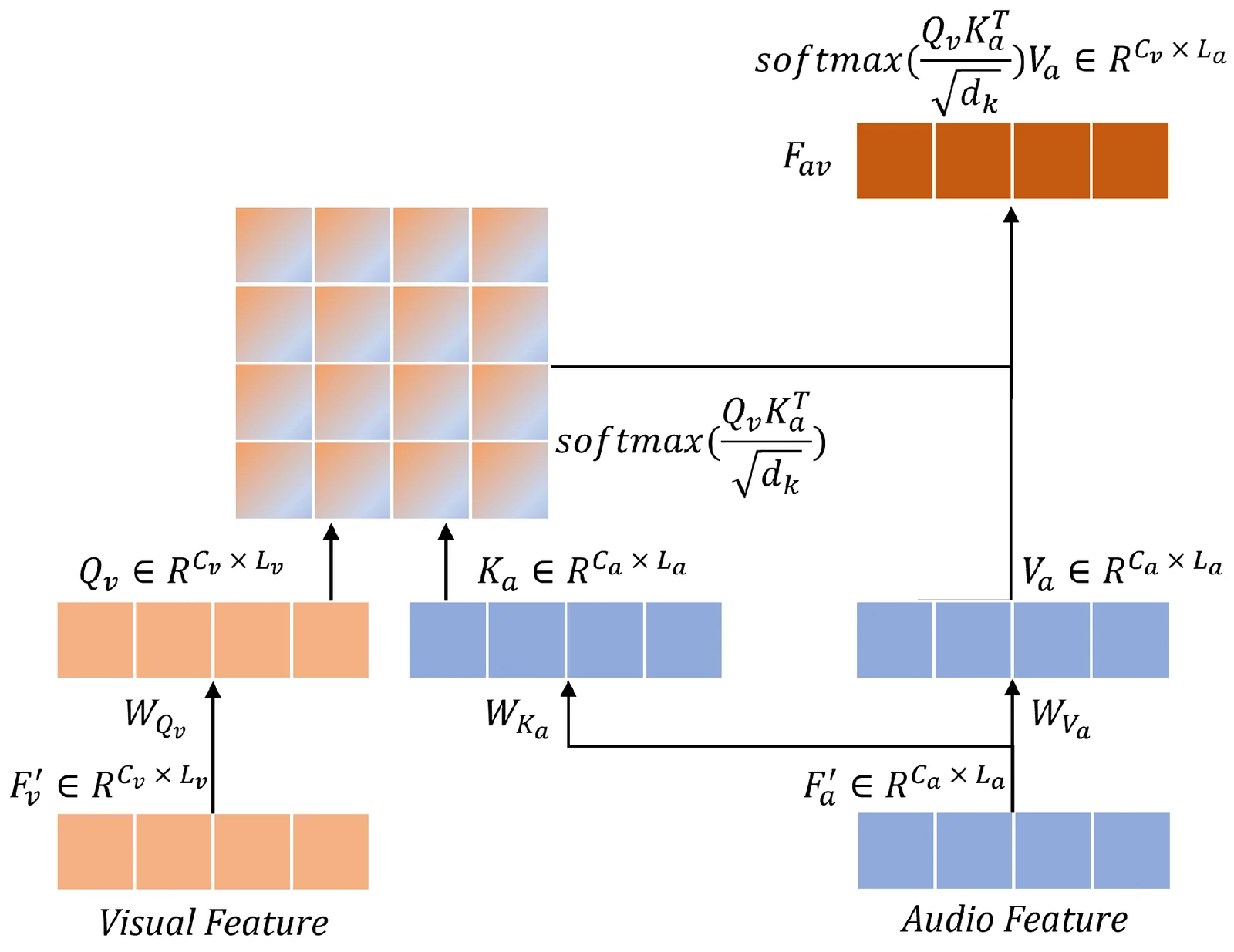
Structure of the cross-modal audio–visual attention fusion module.

The CM-AF module (see Figure 3) performs dynamic alignment between visual spatial features and directional audio information. For each visual query $Q_{v}$, it computes attention over audio features:

(4) $$F_{av}=\mathrm{CrossM}\left(F_{v}^{\prime },F_{a}^{\prime }\right)=\mathrm{softmax}\left(\frac{Q_{v}K_{a}^{T}}{\sqrt{d_{k}}}\right)V_{a}$$

This mechanism is crucial in the cabin setting, where multiple concurrent conversations, physical proximity, and speech overlapping with announcement systems require precise multimodal disambiguation.

### 3.4. Prediction Network

The prediction network decodes $F_{av}^{\prime \prime }$ into a dense speaker activity map across time and cabin space. It comprises 3D transposed convolutional layers, Batch Normalization, and ReLU, culminating in a classifier that outputs a 2×T×H×W tensor representing “speaking” and “non-speaking” probabilities per frame.

The speaker probability map is obtained via softmax normalization. Training uses binary cross-entropy loss against annotated speaker masks:

(5) $$Loss_{av}=-\frac{1}{T}\sum_{i=1}^{T}\left({\hat{y}}_{i}\cdot \log\left(y_{i}\right)+\left(1-{\hat{y}}_{i}\right)\cdot \log\left(1-y_{i}\right)\right),$$

where ${\hat{y}}_{i}$ is the model prediction, and $y_{i}$ is the binary ground-truth label for frame *i*.

### 3.5. Cross-Modal Loss for Auxiliary Supervision

To exploit deeper fusion layers, we apply an auxiliary cross-modal loss $Loss_{cm}$ over $F_{av}^{\prime \prime }$ using a shallow prediction head. Predictions are normalized via softmax:

(6) $${\hat{y}}_{i}=\frac{\exp({{\hat{F}}_{av}^{\prime \prime }}_{i})}{\sum_{i=1}^{T}\exp\left({{\hat{F}}_{av}^{\prime \prime }}_{i}\right)}$$

The auxiliary supervision is computed as follows:

(7) $$Loss_{cm}=-\sum_{i=1}^{T}y_{i}\log\left({\hat{y}}_{i}\right)$$

This enhances generalization under partial observability, which is common during in-flight care where visibility and acoustic clarity fluctuate dynamically.

### 3.6. Training Objective

The overall training loss integrates main and auxiliary components:

(8) $$L=Loss_{av}+\lambda Loss_{cm}$$

We use λ=0.2 to emphasize the primary localization objective while regularizing with auxiliary supervision. This composite loss supports stable, low-latency speaker localization under the operational constraints of aviation-based medical assistance.

### 3.7. Evaluation Dataset: AirCabin-ASL

To enable rigorous and context-specific evaluation of our speaker localization framework, we introduce a custom benchmark dataset, AirCabin-ASL(Airborne Cabin Active Speaker Localization). Due to the absence of public datasets tailored for ASD/ASL tasks in aircraft medical environments, AirCabin-ASL is constructed to simulate realistic in-flight emergency interactions under representative cabin conditions.

**Figure 4:**
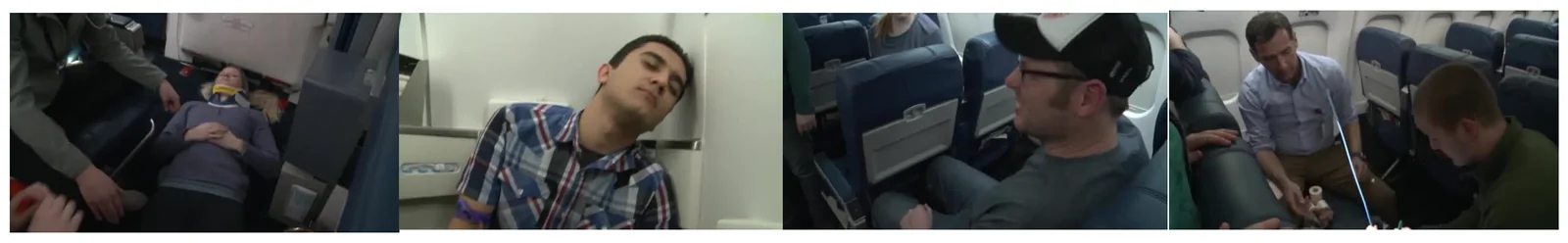
Examples from the AirCabin-ASL dataset.

**Figure 5:**
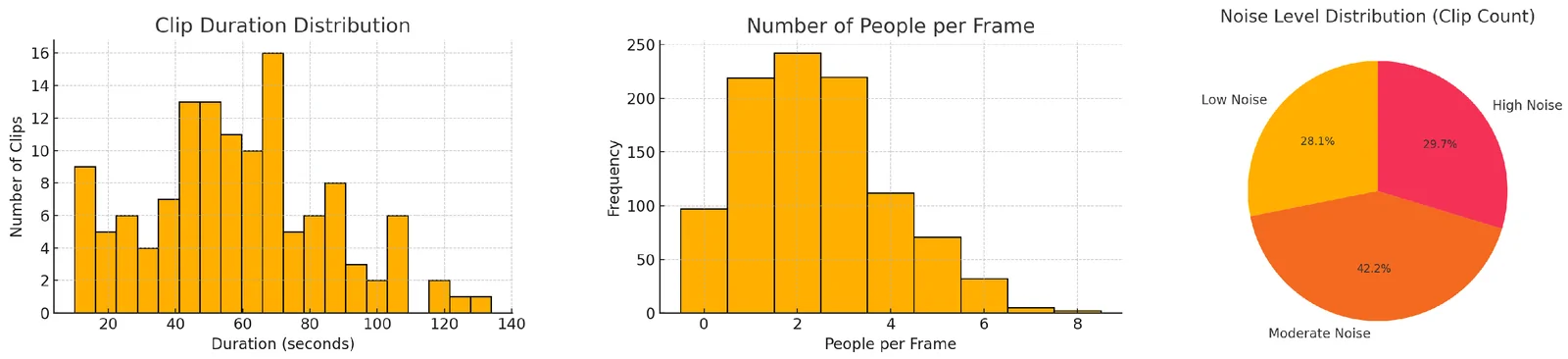
Visual statistics from the AirCabin-ASL dataset. (**Left**): distribution of clip durations; (**Middle**): per-frame person count histogram; (**Right**): noise-level category proportions.

AirCabin-ASL is collected from publicly available online video platforms, including staged medical simulations, aeromedical training videos, and recorded in-flight scenarios. All selected content reflects realistic cabin conditions and was manually reviewed to ensure relevance and quality for evaluation purposes. The dataset contains approximately 5.2 h of multi-channel audiovisual footage, segmented into 6 long-form evaluation scenarios and further divided into 128 clips, each ranging from 10 to 180 s in duration. In total, the dataset comprises over 560,000 video frames at 30 FPS, as shown in Figure 4 and Figure 5.

Each frame contains an average of 2.4 visible persons, with at least one labeled face in 96.2% of frames. The mean number of active speakers per frame is 1.1, reflecting a mixture of monologue and overlapping dialogue patterns. Occlusions (e.g., due to oxygen masks, seatbacks, or passenger movement) are present in approximately 27% of all face instances.

The clips are manually categorized into noise-level conditions: 28% low-noise, 42% moderate-noise, an 30% high-noise based on estimated SNR and background activity. Both fixed and handheld camera views are included to simulate real-world surveillance and crew-held perspectives.

All video samples are manually annotated at the frame level to support precise speaker localization evaluation. Annotations include active speaker labels, face bounding boxes with occlusion indicators (e.g., for oxygen masks or hats), speaker identity across time, and temporal alignment with corresponding multi-channel audio. Basic preprocessing—such as audio–video synchronization, spectral noise suppression, and face tracking via a YOLOv8 + DeepSORT pipeline—is applied to enhance input quality.

## 4. Experiments

### 4.1. Datasets

To support model training and evaluation in a scalable manner, we adopt a hybrid dataset strategy. We leverage two publicly available audio–visual datasets—AVA-ActiveSpeaker [9] and EasyCom [10]—for both training and intermediate validation, while reserving our domain-specific AirCabin-ASL dataset exclusively for final evaluation.

AVA-ActiveSpeaker provides over 3.65 million labelled frames sourced from movies and documentary footage. Despite its open-domain nature, its diversity in speaker identity, viewpoint variation, and acoustic environments offers a strong foundation for learning speaker–visual correspondence.

EasyCom contributes audio–visual recordings from reverberant indoor settings using microphone arrays. Its coverage of multi-party conversations, overlapping speech, and occluded faces closely resembles the interaction complexity encountered in enclosed aircraft cabins.

By using AVA and EasyCom for model development, and employing AirCabin-ASL solely for benchmarking, we ensure that evaluation results reflect true generalization to aviation-specific, high-stakes environments. This separation also minimizes dataset bias and simulates realistic deployment conditions for onboard speaker localization systems.

### 4.2. Experimental Setup and Evaluation Metrics

The input dimensions for the visual and audio modalities are 3×8×224×224 and 2×16×224×224, respectively, with audio features obtained via Short-Time Fourier Transform (STFT). Each modality uses a 3D ResNet pre-trained 200 times on the KM dataset as an encoder [31], and the entire model is implemented using PyTorch 2.4.1. Training hyperparameters (optimizer, learning rate, and batch size) remain consistent with prior settings.

Previously, Mean Average Precision (mAP) was the sole evaluation metric. While effective in measuring detection performance, it offers limited insight into the spatial localization quality. To address this, we introduce an additional metric:

- Mean Average Precision (mAP): This evaluates the match between predicted speaker locations and ground truth across video frames. Higher values indicate better detection precision.
- Mean Intersection over Union (mIoU): This measures the spatial alignment by computing the average IoU between predicted and ground-truth bounding boxes:
  (9) $$mIoU=\frac{1}{N}\sum_{i=1}^{N}\frac{A_{i}^{\mathrm{pred}}\cap A_{i}^{\mathrm{gt}}}{A_{i}^{\mathrm{pred}}\cup A_{i}^{\mathrm{gt}}}$$
  where *N* is the number of test samples. Predictions with IoU > 0.5 are considered true positives. A higher mIoU indicates more accurate spatial localization.

By jointly employing mAP and mIoU, we assess both detection performance and spatial precision, enabling a more comprehensive evaluation of the model.

### 4.3. Experimental Results and Analysis

To evaluate the effectiveness of the proposed Cross-Modal Audio–Visual Fusion Network (CMAVFN) in the context of active speaker localization (ASL) for in-flight medical support scenarios, we conduct comprehensive experiments using the custom-built AirCabin-ASL dataset. Additional comparative evaluations are performed on the AVA-ActiveSpeaker [9] and EasyCom [10] benchmarks to assess generalization and cross-domain performance.

#### 4.3.1. Experiments on the AVA-ActiveSpeaker Dataset

**Comparison with Other Methods.** The comparison results between the proposed attention-based cross-modal active speaker localization model and other approaches on the AVA-ActiveSpeaker dataset are presented in Table 1. The experimental results demonstrate that the proposed model outperforms existing mainstream algorithms on the speaker localization task. Compared with the audio–visual fusion model introduced in the previous paper, our method improves localization accuracy (mAP) by 0.67%, and achieves a significant improvement of 2.40% in the newly introduced mIoU. These results further confirm the effectiveness and superiority of the proposed cross-modal audio–visual fusion method in enhancing speaker localization precision.

**Ablation Study on Feature Fusion Methods.** To validate the design of the cross-modal fusion module in the proposed network (as shown in the left half of Figure 2), we adopt a three-branch structure: visual multi-head attention ($F_{vself}^{\prime }$), visual-only branch ($F_{v}^{\prime }$), and audio–visual cross-modal attention ($F_{avcross}^{\prime }$). In the ablation study, the $F_{v}^{\prime }$ branch is first removed, and then the remaining two branches are fused using either concatenation (Cat) or addition (Add). Subsequently, the visual branch is added back to evaluate its contribution. The experimental settings remain the same. Results are shown in Table 2.

The results indicate that, when only using $F_{vself}^{\prime }$ and $F_{avcross}^{\prime }$, the Add operation outperforms Cat, improving mAP by 2.63% and mIoU by 6.68%, while reducing the number of parameters. Furthermore, adding back the visual-only branch $F_{v}^{\prime }$ yields additional gains (mAP ↑ 0.13%, mIoU ↑ 0.91%), highlighting the importance of visual features in speaker localization.

**Ablation Study on Cross-Modal Fusion Direction.** To analyze the effect of fusion direction in the cross-modal module, we keep the visual attention ($F_{vself}^{\prime }$) and visual branch ($F_{v}^{\prime }$) unchanged, and only vary the direction of cross-modal attention: audio-to-visual ($F_{avcross}^{\prime }$) vs. visual-to-audio ($F_{vacross}^{\prime }$). The rest of the experimental setup remains fixed. Results are shown in Table 3.

Audio-to-visual fusion achieves better performance than visual-to-audio fusion, improving the mAP by 1.84% and mIoU by 4.79%. This confirms the dominant role of visual features in localization and the effectiveness of enhancing them with audio cues.

**Ablation Study on the Number of Self-Attention Blocks.** This section evaluates how the number of self-attention blocks (N) affects performance. The setup uses Add fusion and audio-to-visual cross-modal attention. Only the number of self-attention blocks on the right of Figure 2 varies. Results are shown in Table 4.

Performance improves as the number of self-attention blocks increases up to 5, with peak values at Self-5. Beyond this, performance slightly drops, suggesting potential overfitting or computational redundancy.

**Ablation Study on Cross-Modal Loss and Hyperparameter λ.** Finally, we assess the effectiveness of the auxiliary cross-modal loss function and hyperparameter λ. The base configuration uses Add fusion, audio-to-visual fusion, and five self-attention blocks. Results are shown in Table 5.

Adding the cross-modal loss consistently improves the model performance. The best results are obtained with λ=0.2, improving the mAP by 0.50% and the mIoU by 0.17% over the version without $Loss_{cm}$. However, it also increases the parameter count due to the additional prediction module. This validates the auxiliary loss’s role in enhancing feature alignment and boosting localization accuracy.

**Ablation Study on Audio–Visual Modalities.** To investigate the contribution of audio features in the proposed CMAVFN, which leverages a diffusion-inspired architecture for enhanced feature fusion, we conduct an ablation study comparing a visual-only model against the full audio–visual fusion model. In the visual-only configuration (*Visual*), the audio–visual cross-modal attention ($F_{avcross}^{\prime }$) is replaced with an additional visual self-attention mechanism ($F_{vself}^{\prime }$), effectively removing audio input ($F_{a}^{\prime }$). The full model (*Audio + Visual*) uses the complete architecture with audio–visual cross-modal attention, as described in Section 3.2. Results are presented in Table 6.

The results demonstrate that the visual-only model achieves a competitive mAP of 95.84%, surpassing several baselines in Table 1 (e.g., MuSED at 95.6%). The full audio–visual model outperforms the visual-only configuration, improving the mAP by 1.02% and the mIoU by 1.82%, maintaining a robust performance across diverse scenes. The lower mIoU of the visual-only model (71.83% vs. 73.65%) suggests that audio features enhance the localization precision by providing complementary cues, particularly in scenarios with ambiguous visual information (e.g., partial face visibility or low lighting). These findings highlight the critical role of visual features in speaker localization while confirming that audio–visual fusion significantly enhances both accuracy and precision, aligning with the demands of in-flight medical support applications where robust localization is essential.

#### 4.3.2. Experiments on the Easycom Dataset

**Comparative experiments and cross-modal loss ablation study on the EasyCom dataset.** In addition to the AVA-ActiveSpeaker dataset mentioned above, this paper presents comparative experiment results of the proposed model and an ablation study on the cross-modal loss using a 6-channel audio configuration, which aligns with common aircraft cabin microphone arrays, on the EasyCom dataset. The experimental setup is consistent with the AVA-ActiveSpeaker experiments. Detailed results are shown in Table 7.

Specifically, compared with the results without the cross-modal loss function ($Loss_{cm}$), our method with a 6-channel audio configuration improves the localization accuracy by 0.22% and the localization precision by 0.56%, demonstrating the effectiveness of the proposed algorithm in enhancing the localization precision. This also fully illustrates that the cross-modal attention mechanism can effectively enhance the interaction between different modalities, leveraging the rich spatial cues provided by the 6-channel audio setup. The model performance is optimized with the incorporation of the cross-modal loss function (with $0.2\times Loss_{cm}$), achieving the best results: a localization accuracy of 95.12% and a localization precision of 56.87%.

**Ablation study of the number of audio channels.** To verify the impact of multichannel audio on the model proposed in this paper, an ablation experiment on the number of audio channels is conducted on the EasyCom dataset. Other configurations remained unchanged, and the number of audio channels (N-channel) is set to 2, 4, and 6, respectively, to reflect configurations commonly used in aircraft cabin microphone arrays. Experimental results are shown in Table 8.

The results indicate that on the EasyCom dataset, using 4-channel audio data, outperforms 2-channel audio data, with the mAP improving by 1.44% and the mIoU increasing by 2.51%. Incorporating a 6-channel configuration, which aligns with common aircraft cabin microphone arrays, further enhances the performance, yielding an additional 0.74% improvement in the mAP and 0.94% in the mIoU compared to the 4-channel setup. This validates the effectiveness of multichannel audio data in improving the accuracy and precision of speaker localization, as additional channels provide richer spatial cues for disambiguating speakers in acoustically complex environments.

However, the localization precision (mIoU) on the EasyCom dataset remains significantly lower than that on the AVA-ActiveSpeaker dataset (73.65%). The primary reason for this gap is the extremely small facial area in the EasyCom dataset, where faces typically occupy less than 1% of the image, compared to a much higher proportion in the AVA-ActiveSpeaker dataset. This small facial area, due to the wide field of view in egocentric cabin recordings, makes it challenging for the model to extract precise visual features, leading to difficulties in aligning predicted bounding boxes with ground-truth annotations.

#### 4.3.3. Experiments on the AirCabin-ASL Dataset

To evaluate CMAVFN’s robustness in aircraft cabin environments, we assess models pretrained on AVA-ActiveSpeaker and EasyCom using the AirCabin-ASL dataset without domain-specific fine-tuning. We segment AirCabin-ASL into three noise-level subsets: **Low Noise** (e.g., mid-flight check-ins with ambient silence), **Moderate Noise** (e.g., near-galley conversations with engine hum), and **High Noise** (e.g., turbulence, overhead announcements, or multi-party urgency). Table 9 summarizes the performance under these varying conditions.

Models pretrained on AVA consistently outperform those trained on EasyCom across all noise categories, achieving an overall mAP of **91.35%** and mIoU of **64.77%**. The advantage stems from AVA’s broader diversity in visual perspectives and vocal expressions, which generalize better to the constrained and cluttered aircraft cabin setting. The EasyCom-pretrained model, while slightly less effective, still achieves a robust overall mAP of **89.77%** and mIoU of **63.81%**, benefiting from its egocentric cabin-specific pretraining. As expected, the performance declines under high-noise conditions due to overlapping commands, background announcements, and engine resonance.

Despite these challenges, both models exhibit strong zero-shot generalization, confirming CMAVFN’s capacity to handle non-frontal viewpoints, partial face visibility (e.g., oxygen masks, head tilts), and reverberant in-flight acoustics. These results validate the model’s applicability to real-time speaker localization in in-flight medical scenarios, where clear and timely verbal coordination is critical.

**Figure 6:**
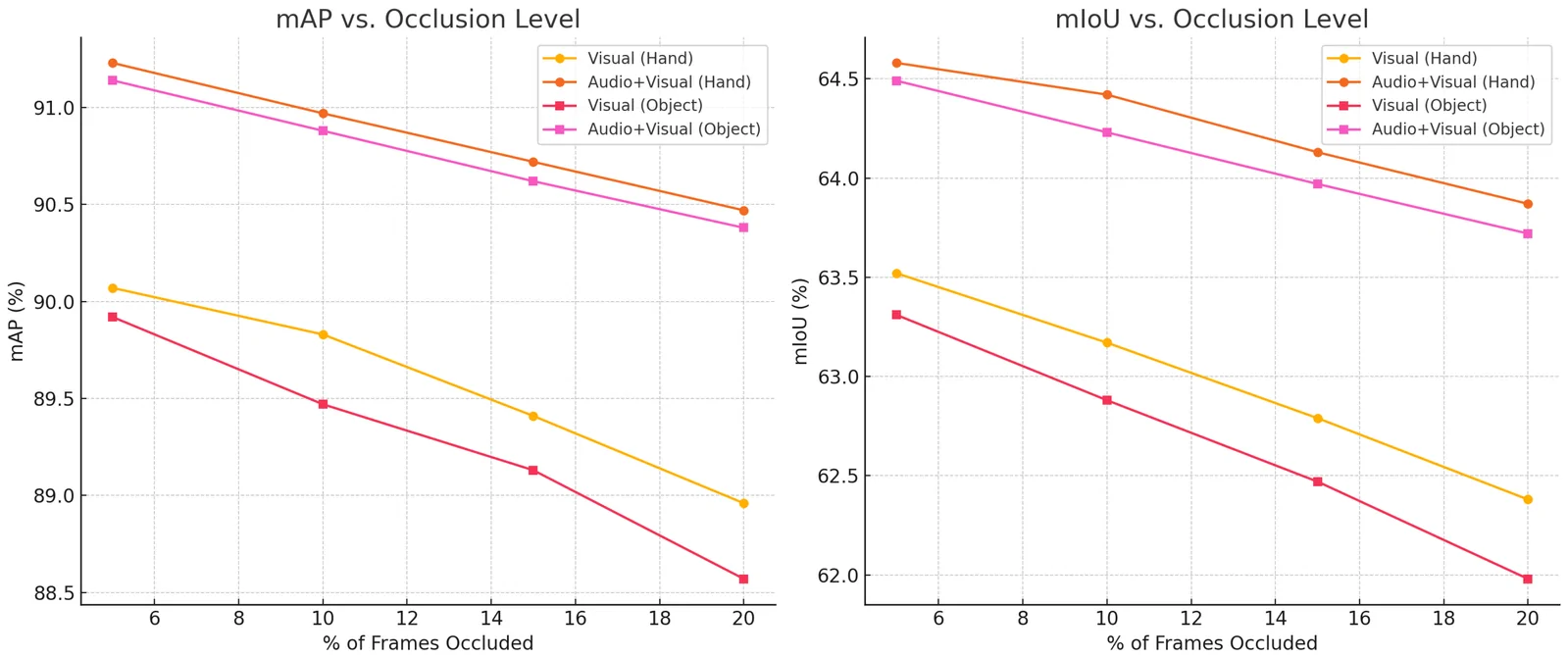
Robustness to visual occlusions on the AirCabin-ASL Dataset.

**Robustness to Visual Occlusions.** To evaluate CMAVFN’s robustness under visual occlusions, which are common in in-flight medical contexts (e.g., hand gestures or medical equipment blocking faces), we synthetically apply occlusions to 5%, 10%, 15%, and 20% of the bounding boxes in the AirCabin-ASL test set. Two occlusion types are examined: hand occlusions (mimicking crew or passenger interactions) and object occlusions (simulating oxygen masks or onboard medical tools). We compare Visual-only and Audio+Visual models pretrained on AVA-ActiveSpeaker, with results shown in Figure 6.

**Figure 7:**
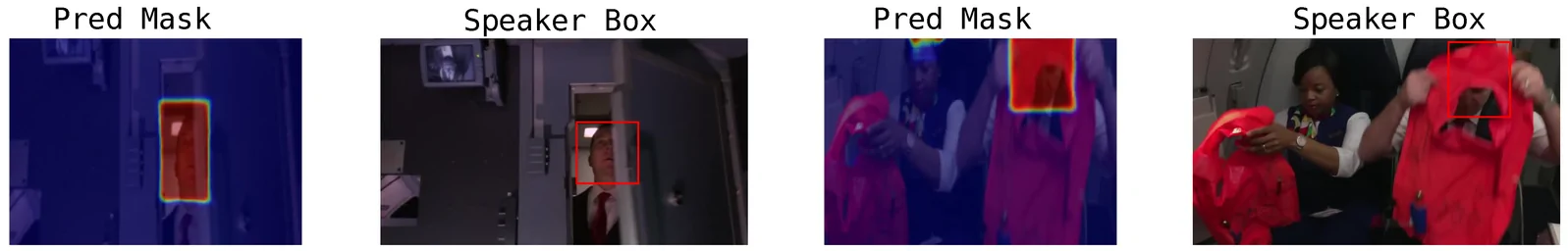
Visualization results of AirCabin-ASL dataset in visual occlusion scenarios.

Across all occlusion levels and types, the Audio+Visual model consistently outperforms the visual-only baseline. Despite some fluctuations across occlusion levels—reflecting the realistic variance of occlusion scenarios—the Audio+Visual model achieves mAP gains generally ranging from 1.10% to 1.70% and mIoU gains from 1.20% to 1.60%. Notably, the performance advantage persists even at the highest occlusion level (20%), where the Audio+Visual model maintains a strong performance (e.g., 90.47% mAP and 63.87% mIoU under hand occlusion), whereas the visual-only model sees a sharper decline (e.g., down to 62.38% mIoU). It is also shown in Figure 7 that our model can still localize stably when more than 50% of the speaker’s facial region is occluded by the model.

While the results exhibit some non-monotonic behaviour—such as occasional metric rebounds at 10% or 15% occlusion levels—these are consistent with the expected variability in face detection accuracy under partially occluded and dynamically changing scenes. Overall, object occlusions tend to produce slightly more degradation than hand occlusions, likely due to their irregular coverage of key facial regions, aligning with real-world cases where medical devices obscure landmarks critical for visual understanding.

#### 4.3.4. Visualization Examples

**Figure 8:**
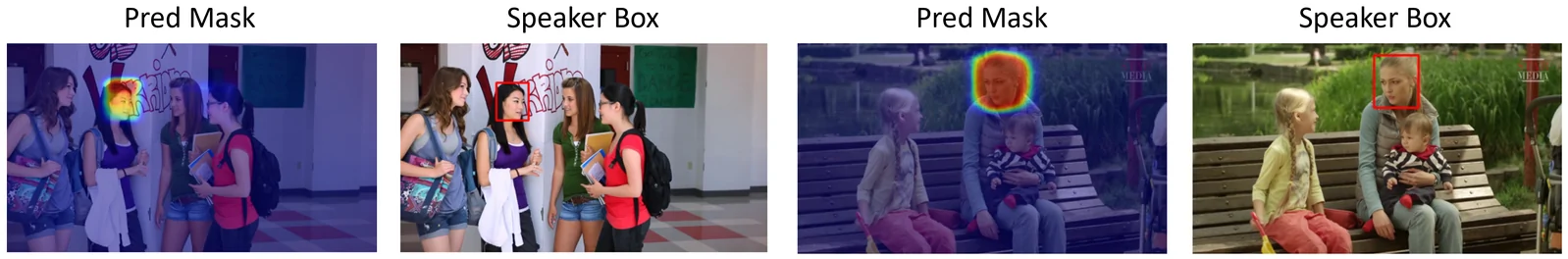
Visualization results on the AVA-ActiveSpeaker dataset.

**Figure 9:**
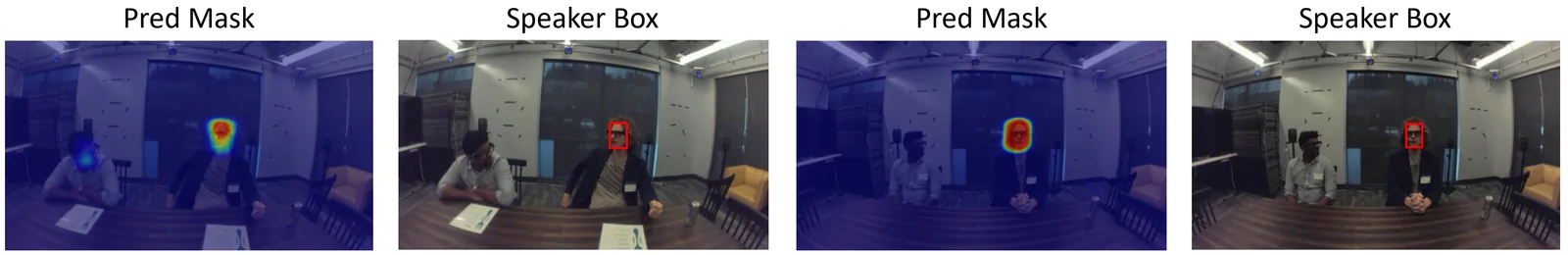
Visualization results on the EasyCom dataset.

**Figure 10:**
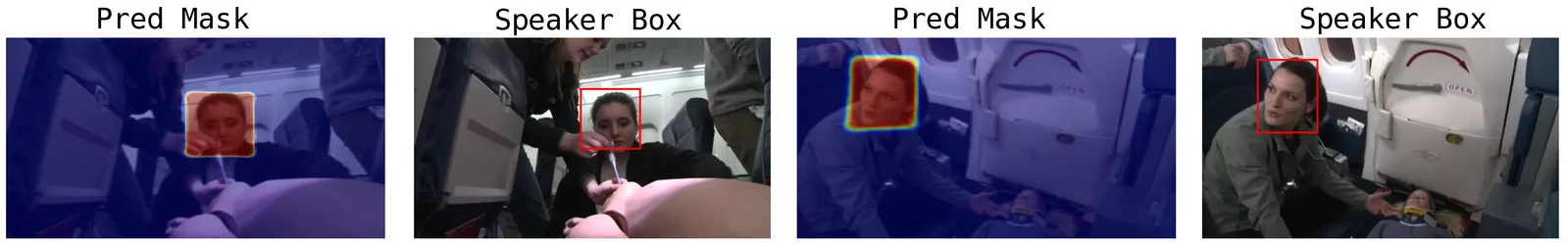
Visualization results on the AirCabin-ASL dataset.

Figure 8, Figure 9 and Figure 10 present qualitative results of our model on the AVA-ActiveSpeaker, EasyCom, and AirCabin-ASL datasets, respectively. Each example includes the predicted attention heatmap (“Pred Mask”) and the ground-truth (“Speaker Box”).

On AVA-ActiveSpeaker, the model effectively captures speaker cues in diverse visual settings with clear frontal faces and consistent lighting. EasyCom results demonstrate robust performance in reverberant and cluttered indoor environments, with accurate speaker localization even under occlusion and multi-speaker interactions.

In the AirCabin-ASL dataset, which features constrained viewpoints and aviation-specific challenges (e.g., occlusion from seatbacks, masks, and non-frontal angles), the model maintains a strong localization performance. Notably, despite elevated background noise and partial visibility, predicted speaker regions remain well-aligned with ground truth annotations. These visualizations highlight the model’s generalization capability across domains, and underscore its effectiveness in high-stakes environments, such as in-flight medical response.

#### 4.3.5. Limitations

While CMAVFN achieves state-of-the-art performance, several limitations remain. First, the model contains approximately 115M parameters, which increase the memory footprint and pose challenges for deployment on resource-constrained devices. Second, although our implementation achieves real-time inference at 50 fps on an NVIDIA GeForce RTX 4090 GPU (NVIDIA Corporation, Santa Clara, CA, USA), maintaining this speed on embedded or low-power platforms may require further model compression or optimization. Third, the performance still degrades under extreme conditions, such as faces occupying less than 1% of the frame or high-noise scenarios with overlapping speech, where the localization accuracy drops by 3–5%. Addressing these issues through lightweight architectures and advanced noise-robust strategies will be key directions for future work.

## 5. Conclusions

In this paper, we introduce an end-to-end audio–visual active speaker localization framework tailored for the acoustically and visually constrained environments encountered in aircraft cabins during in-flight medical scenarios. Our Cross-Modal Audio-Visual Fusion Network (CMAVFN) integrates spatially aligned visual features with directional multi-channel audio inputs via a novel cross-modal attention mechanism, effectively modeling complex interactions between speech activity and environmental conditions, such as engine noise, cabin announcements, passenger density, and visual occlusion from medical equipment or seating arrangements. To improve the spatial localization accuracy under such conditions, we incorporate an auxiliary cross-modal supervision loss that enhances representation learning beyond standard cross-entropy objectives. Our use of the mean Intersection over Union (mIoU) as a spatially aware evaluation metric ensures a fine-grained assessment of localization quality in confined and occlusion-prone cabin layouts. Extensive experiments on our custom AirCabin-ASL dataset, along with evaluations on AVA-ActiveSpeaker and EasyCom, confirm the superiority of our framework in both mAP and mIoU metrics. Ablation studies further highlight the impact of key architectural innovations, including asymmetric attention fusion, spatial normalization, and multi-level loss design. Our findings demonstrate the robustness and practical viability of CMAVFN in real-time speaker localization under realistic aviation constraints. This work lays a technical foundation for future deployment in intelligent in-flight medical systems, including automated medical interaction logs, post-event communication retrieval, and onboard patient-monitoring support in commercial or emergency aviation contexts.

## Figures and Tables

**Table 1:** AVA-ActiveSpeaker.

Method	mAP (%)	mIoU (%)	Params (M)
ASC [32]	87.1	-	23.5
MAAS [27]	88.8	-	22.5
TalkNet [5]	92.3	-	15.7
ADENet [19]	93.2	-	33.2
ASDNet [33]	93.5	-	51.3
SPELL [34]	94.2	-	22.5
SPELL+ [34]	94.9	-	>45.0
LoCoNet [35]	95.2	-	-
MuSED [36]	95.6	-	16.1
**Ours**	**96.86**	**73.65**	115.19

**Table 2:** Ablation results on feature fusion methods on the AVA-ActiveSpeaker dataset.

Feature Jointing	Branches	mAP (%)	mIoU (%)
Cat	$F_{vself}^{\prime }$, $F_{avcross}^{\prime }$	93.60	65.89
Add	$F_{vself}^{\prime }$, $F_{avcross}^{\prime }$	96.23	72.57
**Add**	$F_{\mathrm{vself}}^{\prime }$, $F_{v}^{\prime }$, $F_{\mathrm{avcross}}^{\prime }$	**96.36**	**73.48**

**Table 3:** Ablation results on cross-modal fusion direction on the AVA-ActiveSpeaker dataset.

Cross-Modal Direction	mAP (%)	mIoU (%)
$F_{vacross}^{\prime }$	94.52	68.69
$F_{\mathrm{avcross}}^{\prime }$	**96.36**	**73.48**

**Table 4:** Ablation results on the number of self-attention modules on the AVA-ActiveSpeaker dataset.

Attention-Block N	mAP (%)	mIoU (%)
Cross-1 + Self-3	96.02	71.61
Cross-1 + Self-4	96.34	72.94
**Cross-1 + Self-5**	**96.36**	**73.48**
Cross-1 + Self-6	96.22	72.65
Cross-1 + Self-7	96.11	72.63

**Table 5:** Ablation on cross-modal loss and λ on the AVA-ActiveSpeaker dataset.

Loss	mAP (%)	mIoU (%)
w/o $Loss_{cm}$	96.36	73.48
w/0.1 × $Loss_{cm}$	96.59	73.61
**w/0.2 × ${\mathrm{Loss}}_{\mathrm{cm}}$**	**96.86**	**73.65**
w/0.3 × $Loss_{cm}$	96.40	73.55

**Table 6:** Ablation results of audio–visual modalities on the AVA-ActiveSpeaker dataset.

Model	mAP (%)	mIoU (%)
Visual	95.84	71.83
**Audio + Visual**	**96.86**	**73.65**

**Table 7:** Comparative experiments and cross-modal loss ablation results on the Easycom dataset.

Model	mAP (%)	mIoU (%)
w/o $Loss_{cm}$	94.90	56.31
**w/$0.2\times {\mathrm{Loss}}_{\mathrm{cm}}$**	**95.12**	**56.87**

**Table 8:** Ablation results of audio channel number on the Easycom dataset.

N-Channel	mAP (%)	mIoU (%)
N = 2	92.94	53.42
N = 4	94.38	55.93
**N = 6**	**95.12**	**56.87**

**Table 9:** Performance on AirCabin-ASL dataset under varying noise conditions.

Training Dataset	Noise Level	mAP (%)	mIoU (%)
AVA-ActiveSpeaker	Low Noise	93.78	68.91
	Moderate Noise	91.84	65.13
	High Noise	88.42	60.27
	**Overall**	**91.35**	**64.77**
EasyCom	Low Noise	92.66	67.82
	Moderate Noise	90.09	64.39
	High Noise	86.57	59.21
	**Overall**	**89.77**	**63.81**

## Data Availability

The original contributions presented in this study are included in the article. Further inquiries can be directed to the corresponding author.
